# Sublethal Effects of α-Cypermethrin on the Behavioral Asymmetries and Mating Success of *Alphitobius diaperinus*

**DOI:** 10.3390/insects15100804

**Published:** 2024-10-15

**Authors:** Demeter Lorentha S. Gidari, Nickolas G. Kavallieratos, Maria C. Boukouvala

**Affiliations:** Laboratory of Agricultural Zoology and Entomology, Faculty of Crop Science, Agricultural University of Athens, 75 Iera Odos Str., 11855 Athens, Greece; dlgidari@aua.gr (D.L.S.G.); mbouk@aua.gr (M.C.B.)

**Keywords:** stored-product insect, courtship, mating behavior, laterality, sublethal concentrations, pyrethroid insecticide, *Alphitobius diaperinus*

## Abstract

**Simple Summary:**

This study investigated the behavioral asymmetries and mating success of *Alphitobius diaperinus* (Panzer) (Coleoptera: Tenebrionidae) under sublethal exposures to α-cypermethrin. Control males exhibited a right-side bias, which increased the likelihood of successful copulation, especially when head butts preceded mounting. A similar pattern was observed for LC_10_-exposed males, though their success rate slightly declined. In females, the right-side approach also enhanced copulation success. The direction of approach and mounting influenced the time required for key mating behaviors like mate detection and mounting. Males exposed to LC_30_ of α-cypermethrin needed a longer time for mate detection compared to control and LC_10_, especially when approaching from the back or front side. Females of the LC_30_ group were also influenced, with shorter copulation times when approaching from the right. These findings demonstrate that α-cypermethrin affects both mating success and the duration of related behaviors.

**Abstract:**

Sublethal exposure to insecticides can adversely impact various biological and behavioral characteristics of insects. Although α-cypermethrin has been previously tested for its effects on control of *Alphitobius diaperinus*, there is no knowledge about the effect of this insecticide on its behavioral asymmetries and mating success. Μales at all exposures (control, LC_10_, and LC_30_), that first approached their mate, showed right-biased tendency (approached their mate from their right side) in mate recognition. Females, however, showed variation in this behavior between the three exposures. Right-biased tendency of males in all treatment scenarios led to a higher percentage of successful copulations compared to the three other directions. For males that first approached their mate, the insecticide did not affect their lateralization of the first approach but did affect their copulation success. The duration of copulation time was reduced after the exposure to the insecticide, with the longest duration noted in the control females (63.0 s) and the lowest in the α-cypermethrin LC_30_ females (46.9 s). Moreover, at the α-cypermethrin LC_10_ exposure, mate recognition time was reduced, as opposed to α-cypermethrin LC_30_ exposure where mate recognition time was increased. These results can be further utilized to uncover the behavioral impacts of insecticides, enhancing the effectiveness of pest management in warehouses and poultry production facilities.

## 1. Introduction

*Alphitobius diaperinus* (Panzer) (Coleoptera: Tenebrionidae) is a key species in poultry production and storage facilities [1], as a vector of many dangerous pathogens, such as *Escherichia coli*, turkey coronavirus, Marek’s disease, *Salmonella typhimurium*, and avian leukemia [2,3,4]. These pathogens can also affect humans and cause symptoms such as gastrointestinal disorders and fever [5,6]. The ability of *A. diaperinus* to cause allergic reactions to people who come into frequent contact with this species has also been observed [7]. In intensive poultry production, populations of *A. diaperinus* can reach extremely high levels. For example, around 34.7 million *A. diaperinus* adults, nymphs, and larvae may exist in a poultry production facility [8]. *Alphitobius diaperinus* is a secondary pest of grains, e.g., wheat, rye, barley, flour, bran, and hay [9]. So far, numerous insecticides (both natural and synthetic) have been employed to control *A. diaperinus* larvae or adults [10,11,12,13,14,15,16,17,18].

α-Cypermethrin, a non-systemic pyrethroid (type II) insecticide, is effective through contact and ingestion. It consists of the active isomer of cypermethrin and is highly efficient against a wide range of insect species, e.g., mosquitoes, flies, and other pests commonly found in animal and public housing [19,20,21]. When used according to recommended guidelines and application rates, α-cypermethrin is safe for the general public, occupational exposure, and the environment [22,23]. Despite its efficiency, excessive reliance on α-cypermethrin has led to the development of resistance in various insect pests worldwide [24,25]. This resistance has necessitated higher concentrations, thus increasing control costs and causing negative impacts to the environment and public health [26,27,28]. In addition to causing death in storage insects, synthetic insecticides also produce sublethal effects that can affect their biology and behavior [29,30]. These sublethal effects may include aggressive behavior and adverse impacts on lifespan, fertility, fecundity, courtship, movement, or physical structure [31,32].

Brain lateralization, which refers to the differences in structures and functions between the left and right side of the brain, can improve its capacity to perform cognitive tasks by simultaneously engaging both hemispheres in distinct ways [33]. Lateralization has been investigated in many vertebrates [34,35,36,37,38,39,40,41,42], but in invertebrates and especially insects the knowledge related to this topic is low, except bees [43,44,45,46,47]. Recent studies have concentrated on the laterality behavior of species in the order Coleoptera during mating and courtship, with a particular emphasis on pests of stored products, i.e., Dermestidae, Bostrychidae, Tenebrionidae, Laemophloeidae, Curculionidae, and Silvanidae [48,49,50,51,52,53,54,55]. For *A. diaperinus*, there is limited knowledge about mating behavior and laterality [56,57,58]. Renault [56] revealed that the reproduction success of cold-acclimated *A. diaperinus* adults was superior to that of non-acclimated individuals. Moreover, it is already known that prior sexual experience enables *A. diaperinus* to choose potential mates. Calla-Quispe et al. [57] showed that virgin female adults were significantly more attracted to virgin male adults, while sexually experienced female adults were significantly more attracted to sexually experienced male adults. Although there have beenare former studies of the efficacy of α-cypermethrin against *A. diaperinus* [59], there are no data on the effects of its sublethal concentrations on *A. diaperinus* mating behavior. Therefore, this research piece is focused on assessing the effects of LC_10_ and LC_30_ of α-cypermethrin on *A. diaperinus* asymmetries and mating success.

## 2. Materials and Methods

### 2.1. Alphitobius diaperinus Colonies

Female and male *A. diaperinus* adults were collected from cultures maintained in a mass-rearing facility at the Laboratory of Agricultural Zoology and Entomology, Athens, Greece. The beetles were maintained in continuous darkness, 65% relative humidity (RH), and 30 °C [60]. As a rearing medium, wheat bran and yeast (75% and 25%, respectively), adding apple slices for additional moisture, were used [61].

### 2.2. Sex Recognition

Recognition of males and females was conducted at the pupal stage according to the description of Esquivel et al. [62]. Afterward, pupae of the same age and sex were separately kept under the same conditions, until the beginning of the observations.

### 2.3. Insecticide

For the experiment, the insecticide used was Fendona Top SC (provided by BASF Hellas, Amarousion, Greece). This insecticide contains 1.58% w/w α-cypermethrin active ingredient (a.i.).

### 2.4. Insecticide Contact Toxicity Bioassays

LC_10_, LC_30_, or LC_50_ of α-cypermethrin were determined by impregnating separate pieces of filter paper (Whatman No. 1) with various amounts of the insecticide. This paper was cut in a circular shape to fit in the bottom of the Petri dishes, which had an area of 50.27 cm^2^. A series of α-cypermethrin solutions (0.11, 0.055, 0.0366, 0.0275, and 0.022 mg/cm) in water were prepared. Next, a micropipette was used to spread 1 mL of each α-cypermethrin solution on a different piece of filter paper. Petri dishes with impregnated filter papers were left to dry at 30 °C for 2 h. The same procedure was used to impregnate the control filter papers, with 1 mL distilled water. Within each dish that had been treated with the respective concentration of α-cypermethrin (five dishes per concentration), 20 adults of *A. diaperinus* were released. All dishes remained in continuous darkness in incubators set at 65% RH and 30 °C for 24 h. After that period, the number of dead adults was counted. The aforementioned protocol was repeated twice for both the insecticide and the control. Before starting the behavioral experiments, male and female individuals underwent a 24 h exposure to α-cypermethrin LC_10_ and LC_30_ values, under the same procedure as described above.

### 2.5. Behavioral Experiments

Virgin mature *A. diaperinus* males and females from the control and the group exposed to α-cypermethrin were used. For the observations, a Petri dish arena was used (height: 2 cm and ⌀: 10 cm). A wall made of filter paper was placed around the arena to prohibit any visual signals by the person monitoring the experiments, which may affect the insects’ behavior during observations [48]. Before starting each observation, all individuals were exposed for three hours to the natural conditions of light, which is a sufficient interval for the adjustment of the beetles, as mentioned in previous studies of various stored-products species [49,50,51,52,53,54]. Then, two α-cypermethrin LC_10_-exposed adults of different sexes were introduced in the arena for visual observation of their sexual interaction for 60 min or until the termination of the copulation [49]. During the sexual interaction, if any, the following phases were recorded: (i) time of mate recognition (i.e., the duration from the transfer of the pair to the arena until the male locates the female or conversely), (ii) precopulation (i.e., the time during which the male mounts the female to perform copulation), (iii) copulation success (i.e., the duration from genital contact till genital disengagement), and (iv) total duration of the sexual interaction [48,51,53]. It was also investigated: whether the individual that first approached its mate was male or female and the side of the mate that was first approached (i.e., male or female first approached its mate from the back, front, left, or right side); whether the individuals (male and female) performed headbutts and from which side (back, front, right, or left) the male mounted the female. The same procedure was followed for the control and *A. diaperinus* males and females exposed to α-cypermethrin LC_30_. The numbers of tested pairs of α-cypermethrin LC_10_, LC_30_, and control *A. diaperinus* were 130, 153, and 110 respectively. Pairs that were in proximity to the walls of the arena or did not show any copulation activity for 60 min were discarded [55].

### 2.6. Statistical Analysis

The values of α-cypermethrin that kill 10, 30, and 50% of the exposed individuals, LC_10_, LC_30_, and LC_50_ respectively, were evaluated, with a 95% confidence interval (CI), using probit analysis [63] and the R statistical software (version 2.15.1) [64]. Data analysis of the laterality behavior of *A. diaperinus* was run with the software JMP 16.2 [65]. The effect of orientation when approaching a mate and the lateralization of mounting on the duration of the main behavioral mating traits (mate recognition, mounting, and copula) did not follow a normal distribution and were analyzed using the Steel–Dwass test with a significance level of α = 0.05 [51,53].

## 3. Results

### 3.1. Contact Toxicity on A. diaperinus

Insecticide contact toxicity bioassays revealed that LC_10_, LC_30_, and LC_50_ for *A. diaperinus* were 0.0000666, 0.000120, and 0.000180 mg a.i. cm^−2^, respectively (Table 1).

### 3.2. Impact of α-Cypermethrin on A. diaperinus Mating Behavior and Laterality

**Figure 1:**
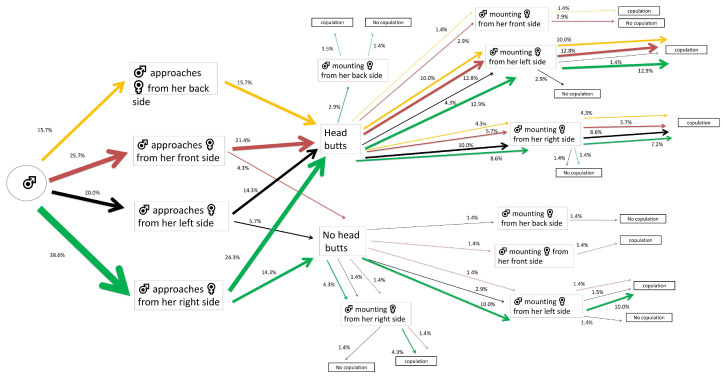
Flow chart of mating and courtship behavior of *Alphitobius diaperinus* male adults exposed to water-impregnated filter paper (control). Different lateralized traits exhibited by the males during each behavioral phase are indicated by the color of the arrows: orange for back-biased, red for front-biased, black for left-biased, and green for right-biased males. The width of each arrow indicates the percentage of insects engaging in each behavior (*n* = 70 pairs).

The majority of the control males that first approached their mates showed a right-side tendency (38.6% out of 100%). Most of these performed head butts (24.3% out of 38.6%) and 12.9% out of 24.3% of males mounted the females from the left side of their bodies, achieving successful copulations (Figure 1).

**Figure 2:**
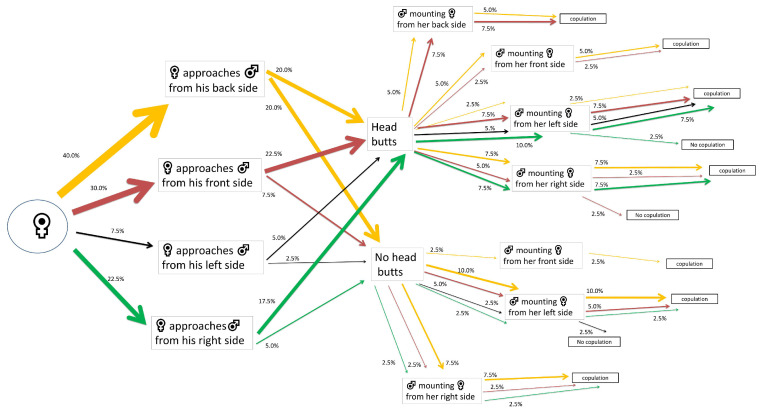
Flow chart of mating behavior and courtship behavior of *Alphitobius diaperinus* female adults exposed to water-impregnated filter paper (control). Different lateralized traits exhibited by the females during each behavioral phase are indicated by the color of the arrows: orange for back-biased, red for front-biased, black for left-biased, and green for right-biased males. The width of each arrow indicates the percentage of insects engaging in each behavior (*n* = 40 pairs).

Most of the control females that approached their mates first exhibited a back-side tendency (40% out of 100%). Half of these (20% out of 40%) performed head butts. A 7.5% out of 20% that performed head butts were mounted by the males from their right side, achieving successful copulations. A 10% out of 20% of females that did not perform head butts were mounted by the males from their left side, achieving successful copulations (Figure 2).

**Figure 3:**
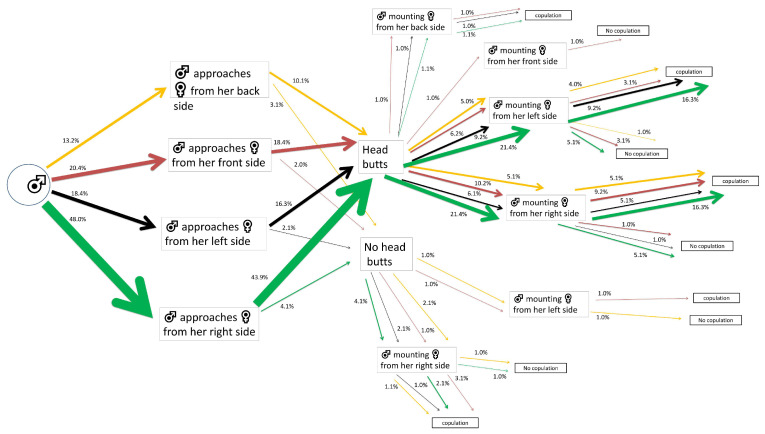
Flow chart of mating and courtship behavior of *Alphitobius diaperinus* male adults exposed to α-cypermethrin LC_10_. Different lateralized traits exhibited by the males during each behavioral phase are indicated by the color of the arrows: orange for back-biased, red for front-biased, black for left-biased, and green for right-biased males. The width of each arrow indicates the percentage of insects engaging in each behavior (*n* = 98 pairs).

A similar pattern to that in the control male group was recorded for α-cypermethrin LC_10_-exposed males that approached their mates first. A 48% out of 100% approached the females from the right side. Almost all of these (43.9% out of 48%) performed head butts. A percentage of 21.4% out of 43.9% mounted the female individuals from their left side, with 16.3% out of 21.4% performing successful copulations. The same percentage of males (i.e., 21.4% out of 43.9%) mounted the females from their right side, achieving 16.3% out of 21.4% mating success (Figure 3).

**Figure 4:**
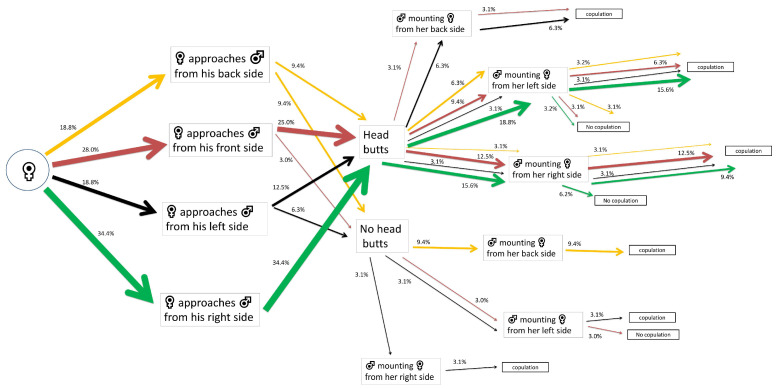
Flow chart of mating and courtship behavior of *Alphitobius diaperinus* female adults exposed to α-cypermethrin LC_10_. Different lateralized traits exhibited by the females during each behavioral phase are indicated by the color of the arrows: orange for back-biased, red for front-biased, black for left-biased, and green for right-biased males. The width of each arrow indicates the percentage of insects engaging in each behavior (*n* = 32 pairs).

For females exposed to α-cypermethrin LC_10_ that approached their mates first, 34.4% out of 100% exhibited a right-side tendency and performed head butts. A percentage of 18.8% out of 34.4% of females were mounted by males from their left side, with 15.6% out of 18.8% performing successful copulations. Fewer females were mounted by males from their right side (15.6% out of 34.4%), achieving only 9.4% out of 15.6% successful mating. (Figure 4).

**Figure 5:**
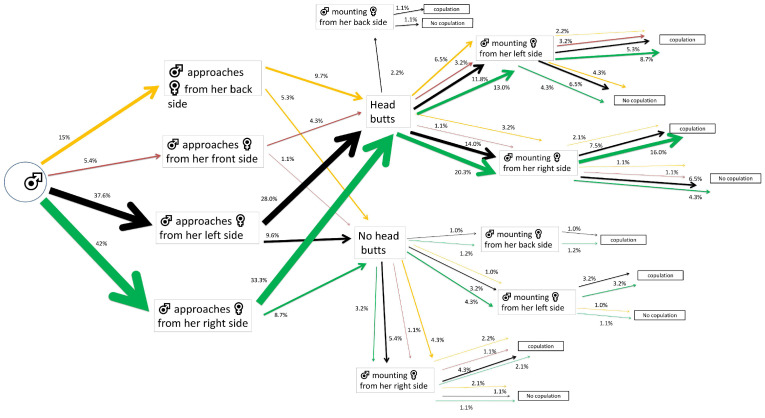
Flow chart of mating and courtship behavior of *Alphitobius diaperinus* male adults exposed to α-cypermethrin LC_30_. Different lateralized traits exhibited by the males during each behavioral phase are indicated by the color of the arrows: orange for back-biased, red for front-biased, black for left-biased, and green for right-biased males. The width of each arrow indicates the percentage of insects engaging in each behavior (*n* = 93 pairs).

As in the two previous cases (LC_10_ and control), α-cypermethrin LC_30_ exposure revealed right-biased tendency (42% out of 100%) for males that approached their mates first. Most of them performed head butts (33.3% out of 42%) and the 20.3% out of this 33.3% mounted females from their right side, with 16% out of this 20.3% achieving successful copulations. The remaining 13% out of 33.3% of males mounted females from their left side, leading to 8.7% out of 13% achieving successful copulations (Figure 5).

**Figure 6:**
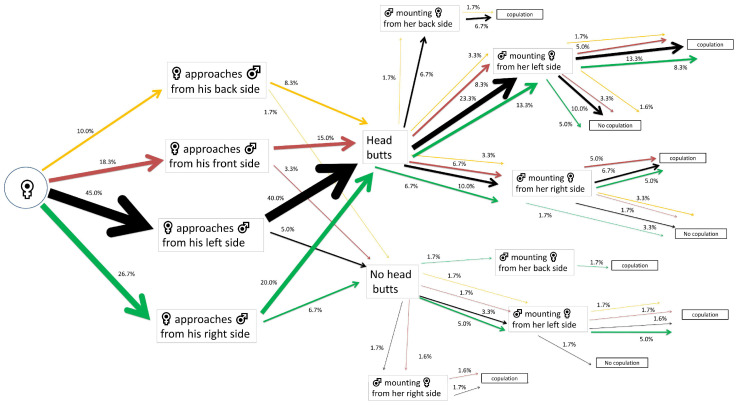
Flow chart of mating and courtship behavior of *Alphitobius diaperinus* female adults exposed to α-cypermethrin LC_30_. Different lateralized traits exhibited by the females during each behavioral phase are indicated by the color of the arrows: orange for back-biased, red for front-biased, black for left-biased, and green for right-biased males. The width of each arrow indicates the percentage of insects engaging in each behavior (*n* = 60 pairs).

α-Cypermethrin LC_30_-exposed females that approached their mates first showed left-biased tendency in 45% out of 100% and the majority of them performed head butts (40% out of 45%). The 23.3% out of 40% were mounted by the males from their left side, leading to 13.3% out of 23.3% successful copulations (Figure 6).

The duration of mating traits of *A. diaperinus* was significantly influenced by the laterality of direction of the first approach and mounting side in all three exposures (control, LC_10_, and LC_30_) (Table 2, Table 3, Table 4 and Table 5). Regarding the effect of the side from which males first approached their mates on the mating traits of *A. diaperinus*, the mate detection duration was significantly higher for all directions of the LC_30_-exposed males and for left-biased control males (338.4 s for back-, 435.6 s for front-, 366.3 s for left-, and 355.4 s for right-biased male individuals of the LC_30_ exposure and 303.0 s for the left-biased male individuals of the control group, respectively). The shortest mate detection was observed in the back-biased control males (46.1 s) (Table 2).

The effect of the approaching side, when females approached their mates first, caused the lowest and the highest duration of copulation, 46.9 s for the right-biased females exposed to the LC_30_ and 63.0 s for the right-biased females of the control group (Table 3).

This laterality aspect also affected the time needed for the mounting, with the left-biased male individuals of the control group achieving the lowest times of all scenarios tested (287.5 s) (Table 3). In contrast, mounting side laterality, when males approached their mate first, caused the highest time needed for mounting (2139.3 s) in the back-biased males of the LC_30_ exposure (Table 4).

For the LC_30_-exposed males, no front-biased mating traits were recorded, and this was also the case for the copulation direction of the LC_10_ exposed males. Τhe shortest mate detection (15.5 s) was noted in the front-biased females of the control group when they approached their mates first (Table 5). This laterality aspect did not result in any front-biased mating trait for either LC_10_ or LC_30_ exposure.

## 4. Discussion

To date, there has been just one study on the lateralization of *A. diaperinus* mating behavior [58]. The authors examined how directional displacement during the mating behavior of a Peruvian strain (specifically, movements that were either lateralized or non-lateralized) affected both mating success (defined as the occurrence of copulation) and efficiency (measured by the time taken to achieve copulation). The fact that virgin males and females from the Peruvian strain did not show any lateralized approaches in contrast to the tested Greek strain, where males of all exposures showed a right-biased first approach and left-biased mounting approach and females a variety of lateral approaches, is probably due to the different geographical origins. The influence of the geographical origin was also reported in behavioral experiments on Turkish and Czech strains of *Trogoderma granarium* Everts (Coleoptera: Dermestidae), where a higher percentage of males of the Czech strain approached females from the left (53%), while Turkish-strain males exhibited a preference for approaching females from the right (50%) [55].

The findings of this research indicate that laterality significantly influenced the mating success of *A. diaperinus*. Males that first approached their mates from their right side accomplished the highest percentage of successful copulations vs. males that first approached their mates from a different side. Previous studies related to tenebrionids have reported a left-biased tendency. *Tenebrio molitor* L. (Coleoptera: Tenebrionidae) displayed a left-biased preference in recognition and mounting attempts to potential mates which resulted in higher copulation success compared to males that showed a right-side bias [52]. The same tendency was also reported for *Tribolium castaneum* (Herbst) (Coleoptera: Tenebrionidae) and *T. confusum* Jacquelin du Val [48,50]. Earlier studies on other coleopteran families found that right-biased males achieved higher mating success vs. left-biased males. For instance, strains of *Rhyzopertha dominica* (F.) (Coleoptera: Bostrychidae) from three countries (Greece, Romania, and Turkey) demonstrated a preference for right-sided mating, leading to a greater success rate in copulation compared to left-sided and back-side-approaching male individuals [51]. Likewise, *Oryzaephilus surinamensis* (L.) (Coleoptera: Silvanidae) males exhibited a tendency to initiate mating attempts from the right side of female individuals, resulting in higher rates of successful mating compared to left-sided males [53]. However, there are several studies with opposite results, where other stored-product coleopteran pests revealed a left-biased tendency. For example, Romano et al. [48] revealed that *Sitophilus oryzae* (L.) (Coleoptera: Curculionidae) males displayed a preference for left-sided copulation attempts towards potential mates, followed by periodic head wagging biased towards the right. This behavior persisted despite the majority of males initially attempting left-sided copulations.

Based on previous studies, α-cypermethrin is effective against *A. diaperinus* adults, causing 100% mortality after 14 days of exposure at 10% of LC_50_ [59]. However, the impact of sublethal concentrations of this insecticide on *A. diaperinus* is yet to be investigated. According to previous research, low doses of insecticides influence the physiology and behavior of various insect species in different ways [32,66,67]. Kavallieratos et al. [67] studied how chlorantraniliprole affects the movement behavior of *Sitophilus zeamais* Motschulsky (Coleoptera: Curculionidae) and *S. oryzae* at low concentrations. Analysis of their mobility behavior revealed notable differences between chlorantraniliprole-treated specimens and controls, in the absence and presence of food. *Sitophilus oryzae* demonstrated altered movement patterns and fewer approaches to food at sublethal doses, whereas *S. zeamais* presented increased walking time and shorter periods of immobility [67]. Concerning *T. granarium*, the exposure to low chlorfenapyr and pirimiphos-methyl concentrations caused detrimental mobility. Beetles exposed to an LC_30_ concentration of pirimiphos-methyl displayed a noticeably shorter walking duration compared to those exposed to LC_10_ of the tested insecticides, LC_30_ of chlorfenapyr, and control [66]. In the case of insecticides based on plants, the walking and mating behavior of adult *Prostephanus truncatus* (Horn) (Coleoptera: Bostrychidae) was significantly impacted when exposed to the asteraceous hexane extract (HE) of *Acmella oleracea* (L.) R.K. Jansen at LC_10_ and LC_30_ [32]. The current study documents that even low concentrations of α-cypermethrin are able to negatively affect copulation success, reduce the time of copulation, and increase the time needed to recognize the mate. The process of mating is crucial for the expansion of insects as the way to increase their population, become established in new locations, and co-exist with other species [68]. It is already known that an increase in mate recognition time and a reduction in copulation time may lead to unfavorable results for the insect, such as facing issues in their reproduction and population growth [69,70]. Therefore, using smaller quantities of the insecticide, management of *A. diaperinus* is feasible while causing less interruption to the storage and/or poultry environment. Alternative methods to control *A. diaperinus* have been suggested previously. For example, all *A. diaperinus* larvae died when exposed to 40 ppm of O_3_ 36 h post-treatment [71]. Moreover, the use of different strains of *Beauveria bassiana* (Balsamo-Crivelli) Vuillemin (Hypocreales: Cordycipitaceae) resulted in 95% and 62.5% mortality of *A. diaperinus* larvae and adults, respectively, when immersed in a 1 mL suspension containing 10^7^ conidia [72].

This study clearly demonstrated that α-cypermethrin negatively affected the mating process of *A. diaperinus* under sublethal concentrations. Nevertheless, more research is needed to ascertain the effect of α-cypermethrin on insemination success, egg hatching, and offspring morphology. In addition to the management point of view, studying the laterality of mating success may be useful in the *A. diaperinus* mass rearing process, using selected behavioral patterns that enhance the reproduction of this species.

## Figures and Tables

**Table 1:** Contact toxicity of α-cypermethrin on *Alphitobius diaperinus* adults.

Active Ingredient	Unit	LC_10_ (95% CI)	LC_30_ (95% CI)	LC_50_ (95% CI)	*χ*^2^ (df = 23)	*p*
α-cypermethrin	mg a.i./cm^2^	0.0000666 (0.000024–0.000109)	0.000120 (0.0000596–0.000169)	0.000180 (0.000112–0.000229)	10.7	0.986

LC = lethal concentration that kills 10%, 30%, and 50% of the exposed beetles. 95% CI = lower and upper limits of the 95% confidence interval.

**Table 2:** Effect of the approaching side, when males approached their mate first, on the main mating traits of *Alphitobius diaperinus* exposed to impregnated filter paper with water (control), or LC_10_ or LC_30_ of α-cypermethrin.

	Treatment	Direction of the Approach	Behavioral Traits		
**Laterality**			♂ Mate recognition (s)	♂ Mounting (s)	Copulation (s)
	Control	Back	46.1 ± 15.5 ^b^	513.3 ± 80.3 ^f^	57.7 ± 1.9 ^abc^
		Front	123.4 ± 25.4 ^b^	1046.9 ± 140.4 ^e^	58.8 ± 1.0 ^ab^
		Left	303.0 ± 112.6 ^a^	1008.7 ± 156.6 ^ef^	61.5 ± 3.4 ^a^
		Right	108.4 ± 22.1 ^b^	1191.7 ± 121.6 ^de^	60.0 ± 1.5 ^a^
		Tested beetles (*n* = back- + front- + left- + right-biased)	11 + 18 + 14 + 27 = 70	11 + 18 + 14 + 27 = 70	11 + 18 + 8 + 25 = 62
	LC_10_	Back	49.8 ± 18.1 ^b^	1434.1 ± 179.3 ^bcde^	52.1 ± 0.9 ^cd^
		Front	73.4 ± 12.3 ^b^	1120.7 ± 109.3 ^de^	51.7 ± 0.8 ^d^
		Left	88.5 ± 18.5 ^b^	1811.4 ± 159.1 ^ac^	52.2 ± 0.6 ^cd^
		Right	56.7 ± 8.0 ^b^	1438.4 ± 89.2 ^bd^	51.3 ± 0.4 ^d^
		Tested beetles (*n* = back- + front- + left- + right-biased)	13 + 20 + 18 + 47 = 98	13 + 20 + 18 + 47 = 98	10 + 15 + 16 + 36 = 77
	LC_30_	Back	338.4 ± 33.7 ^a^	1768.1 ± 165.0 ^abc^	50.7 ± 0.7 ^cd^
		Front	435.6 ± 60.3 ^a^	1993.4 ± 332.6 ^abc^	51.0 ± 0.7 ^bcd^
		Left	366.3 ± 28.7 ^a^	1878.5 ± 131.8 ^a^	50.4 ± 0.7 ^d^
		Right	355.4 ± 23.9 ^a^	1745.8 ± 94.8 ^ac^	48.3 ± 0.7 ^d^
		Tested beetles (*n* = back- + front- + left- + right-biased)	14 + 5 + 35 + 39 = 93	14 + 5 + 35 + 39 = 93	6 + 4+ 21 + 29 = 60
		*χ*^2^, df, *p*	152.32, 11, <0.001	69.63, 11, <0.001	104.37, 11, <0.001

Values are means (± standard errors). Within each column, different letters indicate significant differences (Steel–Dwass test, *p* < 0.05).

**Table 3:** Effect of the approaching side, when females approached their mate first, on the main mating traits of *Alphitobius diaperinus* exposed to impregnated filter paper with water (control), or LC_10_ or LC_30_ of α-cypermethrin.

	Treatment	Direction of the Approach	Behavioral Traits		
**Laterality**			♀ Mate recognition (s)	♂ Mounting (s)	Copulation (s)
	Control	Back	188.3 ± 76.2 ^ab^	994.9 ± 170.1 ^bc^	57.8 ± 1.4 ^abc^
		Front	68.7 ± 20.8 ^b^	813.9 ± 132.5 ^c^	58.4 ± 1.1 ^ab^
		Left	266.3 ± 157.0 ^ab^	287.5 ± 74.1 ^abc^	54.0 ± 2.0 ^abcd^
		Right	226.4 ± 69.5 ^ab^	1132.7 ± 205.3 ^abc^	63.0 ± 0.8 ^a^
		Tested beetles (*n* = back- + front- + left- + right-biased)	16 + 12 + 3 + 9 = 40	16 + 12 + 3 + 9 = 40	16 + 11 + 2 + 8 = 37
	LC_10_	Back	99.7 ± 39.2 ^ab^	1038.8 ± 165.5 ^abc^	50.8 ± 0.6 ^bcd^
		Front	69.2 ± 19.0 ^b^	1656.1 ± 283.0 ^abc^	51.9 ± 1.4 ^cd^
		Left	121.8 ± 26.1 ^ab^	1203.3 ± 313.5 ^abc^	51.0 ± 1.6 ^cd^
		Right	76.7 ± 17.4 ^b^	1170.0 ± 243.5 ^abc^	51.0 ± 0.7 ^d^
		Tested beetles (*n* = back- + front- + left- + right-biased)	6 + 9 + 6 + 11 = 32	6 + 9 + 6 + 11 = 32	4 + 7 + 5 + 8 = 24
	LC_30_	Back	331.0 ± 81.2 ^ab^	1717.0 ± 354.9 ^abc^	51.3 ± 2.6 ^bcd^
		Front	320.5 ± 52.4 ^a^	1809.9 ± 159.4 ^ab^	49.4 ± 1.6 ^d^
		Left	347.0 ± 22.1 ^a^	1791.6 ± 111.0 ^a^	49.6 ± 0.7 ^d^
		Right	349.1 ± 37.4 ^a^	1716.1 ± 134.9 ^ab^	46.9 ± 1.4 ^d^
		Tested beetles (*n* = back- + front- + left- + right-biased)	6 + 11 + 27 + 16 = 60	6 + 11 + 27 + 16 = 60	3 + 8 + 18 + 12 = 41
		*χ*^2^, df, *p*	152.32, 11, <0.001	69.63, 11, <0.001	57.48, 11, <0.001

Values are means (± standard errors). Within each column, different letters indicate significant differences (Steel–Dwass test, *p* < 0.05).

**Table 4:** Effect of the mounting side, when males approached their mate first, on the main mating traits of *Alphitobius diaperinus* exposed to impregnated filter paper with water (control), or LC_10_ or LC_30_ of α-cypermethrin.

	Treatment	Direction of Mounting	Behavioral Traits		
**Laterality**			♂ Mate recognition (s)	♂ Mounting (s)	Copulation (s)
	Control	Back	490.7 ± 368.5 ^a^	1175.3 ± 51.7 ^abcde^	58.0 ± 0.0 ^ab^
		Front	78.3 ± 41.5 ^bc^	601.0 ± 26.3 ^de^	59.8 ± 2.7 ^a^
		Left	144.6 ± 35.7 ^bc^	982.9 ± 107.6 ^d^	59.4 ± 1.3 ^a^
		Right	104.7 ± 23.4 ^bc^	1100.4 ± 115.0 ^cde^	59.6 ± 1.1 ^a^
		Tested beetles (*n* = back- + front- + left- + right-biased)	3 + 4 + 38 + 25 = 70	3 + 4 + 38 + 25 = 70	1 + 4 + 35 + 22 = 62
	LC_10_	Back	57.3 ± 11.7 ^bc^	1901.7 ± 399.2 ^abcde^	52.3 ± 1.5 ^ab^
		Front	82.2 ± 0.0 ^abc^	762.0 ± 0.0 ^abcde^	-
		Left	51.5 ± 7.7 ^c^	1350.2 ± 83.8 ^bcde^	51.7 ± 0.5 ^b^
		Right	76.5 ± 9.8 ^c^	1504.7 ± 96.5 ^abce^	51.6 ± 0.4 ^b^
		Tested beetles (*n* = back- + front- + left- + right-biased)	3 + 1 + 43 + 51 = 98	3 + 1+ 43 + 51 = 98	3 + 0 + 33 + 41 = 77
	LC_30_	Back	330.5 ± 74.0 ^ab^	2139.3 ± 165.9 ^abcde^	48.3 ± 3.3 ^b^
		Front	-	-	-
		Left	379.4 ± 25.0 ^a^	1874.7 ± 115.9 ^a^	49.7 ± 0.7 ^b^
		Right	363.2 ± 21.5 ^a^	1735.0 ± 91.0 ^ab^	49.3 ± 0.6 ^b^
		Tested beetles (*n* = back- + front- + left- + right-biased)	4 + 0 + 40 + 49 = 93	4 + 0 + 40 + 49 = 93	3 + 0 + 24 + 33 = 60
		*χ*^2^, df, *p*	145.61, 10, <0.001	59.3, 10, <0.001	100.31, 9, <0.001

Values are means (± standard errors). Within each column, different letters indicate significant differences (Steel–Dwass test, *p* < 0.05). Where dashes are shown, no front-biased mounting was observed.

**Table 5:** Effect of the mounting side, when females approached their mate first, on the main mating traits of *Alphitobius diaperinus* exposed to impregnated filter paper with water (control), or LC_10_ or LC_30_ of α-cypermethrin.

	Treatment	Direction of Mounting	Behavioral Traits		
**Laterality**			♀ Mate recognition (s)	♂ Mounting (s)	Copulation (s)
	Control	Back	33.4 ± 12.4 ^c^	463.6 ± 145.5 ^c^	57.2 ± 3.2 ^abc^
		Front	15.5 ± 18.5 ^c^	549.5 ± 28.2 ^c^	59.5 ± 2.5 ^ab^
		Left	191.3 ± 56.1 ^bc^	1143.4 ± 157.7 ^c^	58.1 ± 1.3 ^a^
		Right	230.9 ± 76.8 ^abc^	1061.2 ± 134.8 ^c^	60.5 ± 1.0 ^a^
		Tested beetles (*n* = back- + front- + left- + right-biased)	5 + 4 + 18 + 13 = 40	5 + 4 + 18 + 13 = 40	5 + 4+16+12 = 37
	LC_10_	Back	145.8 ± 40.6 ^abc^	1248.6 ± 367.3 ^abc^	53.4 ± 1.1 ^abcd^
		Front	-	-	-
		Left	65.6 ± 10.1 ^c^	1446.9 ± 212.4 ^abc^	49.9 ± 0.7 ^cd^
		Right	88.4 ± 20.1 ^c^	1132.9 ± 200.4 ^bc^	51.4 ± 0.8 ^bcd^
		Tested beetles (*n* = back- + front- + left- + right-biased)	5 + 0 + 14 + 13 = 32	5 + 0 + 14 + 13 = 32	5 + 0 + 10 + 9 = 24
	LC_30_	Back	287.5 ± 74.1 ^abc^	1606.3 ± 263.2 ^abc^	48.7 ± 2.3 ^d^
		Front	-	-	-
		Left	357.8 ± 24.5 ^a^	1728.8 ± 91.6 ^ab^	49.4 ± 0.8 ^d^
		Right	325.6 ± 27.7 ^ab^	1898.2 ± 145.9 ^a^	48.0 ± 1.2 ^d^
		Tested beetles (*n* = back- + front- + left- + right-biased)	6 + 11 + 27 + 16 = 60	6 + 11 + 27 + 16 = 60	3 + 8 + 18 + 12 = 41
		*χ*^2^, df, *p*	64.17, 9, <0.001	40.99, 9, <0.001	57.04, 9, <0.001

Values are means (± standard errors). Within each column, different letters indicate significant differences (Steel–Dwass test, *p* < 0.05). Where dashes are shown, no front-biased mounting was observed.

## Data Availability

Data is available within the article.
